# Peer Review of “The Exchange of Informational Support in Online Health Communities at the Onset of the COVID-19 Pandemic: Content Analysis”

**DOI:** 10.2196/31416

**Published:** 2021-07-22

**Keywords:** COVID-19, health information, informational support, online health, online health communities, online platform, pandemic, social support

This is a peer-review report submitted for the paper “The Exchange of Informational Support in Online Health Communities at the Onset of the COVID-19 Pandemic: Content Analysis”.

## Round 1 Review

### General Comments

My comments are as follows:

1. The authors [1] did not review relevant existing works carefully. A number of studies on online health communities (OHCs) have been conducted already. You should compare your results with these relevant works.

2. Although you have mentioned that one coder is a limitation, it is an evitable limitation and needs to be overcome, or how can you ensure the accuracy of the results? I suggest that the authors recode the posts and responses by two coders (at least) who are familiar with this field.

3. What are the criteria by which you determine the name of the coding and the definition of the coding?
